# Wanted, More Howards

**Published:** 1889-08-24

**Authors:** 


### WANTED, MORE HOWARDS.

There is a general idea that Howard, "the philanthropist," as we have dubbed him, with our pleasant readiness to give a man a pretty title, reformed our prisons. We appreciate Howard, we like to think of him, for he was a good man, and the existence of such reflects a certain credit on the whole race. We bask in the sunshine of our Howards, as families who are blessed with distinguished relations do in the consciousness of the judge and Sir John. But these families feel that a certain responsibility devolves on them with the distinguished relationship ; they are bound to be at least more genteel than their neighbours. Does the human race at large, do we English in particular, appreciate the responsibility bequeathed us by our Howards ? Do we feel that from the existence of one philanthropist others should spring ; that a good work begun by one man should be carried out and finished by others ? To this the answer must be a qualified negative. Only to a very limited extent is the general public convinced of the duty of carrying on reforms once begun, and there is always a danger of a good object being neglected when it is no longer new as well as good.

With regard to the particular work with which Howard's name is associated, the reforming of prisons, the general idea is that it is satisfactorily done. At meetings devoted to the consideration of the housing of the working classes, excited speakers often declare that our prisons are better than our cottages, and that the burglar and the thief are better lodged than the industrious poor. It may be doubted if there is much truth in the statement; at any rate, it cannot be accepted without grave reservations. The prisons of our large towns are generally healthy; the sanitary conditions of our convict settlements are on the whole satisfactory; but much remains to be done. Fortunately we have an association of gentlemen—the Howard Association—actuated by the spirit of the great philanthropist, who are looking out the dark spots in our prisons and bringing these blots on our system of criminal administration before the notice of the authorities. Fortunately, too, both the present and the late Home Secretaries have shown themselves anxious to bring about all the improvements in their power; but with regard to the places where prisoners are kept before trial, the authority often lies in the hands of the local municipalities, and to move them to action public opinion must be brought to the aid of official commands.

With a view to enlightening public opinion on this subject, the Howard Association have issued a circular stating some of the facts they have met with in the course of their investigations. The condition of the prisons in many towns and the cells in courthouses where assizes are held demands great improvement, and even those severe souls who think that nothing is too bad for the convicted felon will at least admit that some consideration should be shown to those who, though accused, are not yet convicted of crime, and who may be wholly innocent. The report of the association says: "There remain, in some localities, underground places of detention for prisoners awaiting trial, where there is hardly a glimmer of daylight, and in which poor wretches are crowded, perhaps for days, with horrid insanitary arrangements, and this, too, at the time when their lives and liberties are at stake, and should require the accused to have all their faculties about them. This is an abominable state of things. But it actually exists in England at the present time. And meanwhile some of the local authorities who are responsible for it resist injunctions to improve these places, on the score of expense."

It is to a great extent impossible to state in these columns the conditions that obtain in the places alluded to. We must be content to indicate that there are still in our midst houses of detention where no regard is paid to the requirements of common decency, leaving out of question health and propriety. In the City Court of classic Oxford there is "no means of separation of the sexes"; in Hull the accommodation is "deplorably inadequate," and the "constantly crowded cells" are underground, with no access to daylight. At Bodmin, we learn that the prisoners are kept in box-cupboards, 2ft. 6in. long by 1ft. 9in. broad. A prisoner had better by far be placed in the old-fashioned stocks than in one of these cupboards. He would have nearly as much use of his limbs, and at least would be surrounded by the free air of heaven. Such prisons as these are relics of the barbarous ages when a prisoner was held to have forfeited all human rights, and are doubly intolerable when those who are confined in them are not yet proved guilty.

Even in the matter of conveying prisoners to and from the courthouses where they are tried, a great improvement is possible. The regulation prison van, the "Black Maria," may not be an altogether pleasant vehicle, with its dark interior divided into cells; but at least it has the advantage of privacy. Whether guilty or innocent, a man or woman accused of crime for the first time is likely to feel the situation keenly. It is desirable that this wholesome shame should exist, but it is likely to develop into a most unwholesome feeling when the prisoner is exposed to the coarse jests and brutal chaff of his hardened companions. The Home Office committee, appointed to enquire into the matter, report the governor of a large prison as having given it as his opinion that "he thought that any decent man would gladly compound for that ride by a month's imprisonment." The governor of a jail can hardly be a morbidly sensitive character, however fine his feelings may be, and, therefore, such an opinion, coming from such a quarter, has double weight as a testimony against this method of conveyance. Bad as it is for men, it is worse for women. A girl accused, perhaps falsely, of some petty theft, is mixed up, both in the police cell and in the prison van, with the worst of her sex; and in this connection it may be well to recall Lord Tennyson's dictum that men differ "but as heaven and earth; but women, worst and best, as heaven and hell." Apart from the cruelty of such treatment, it must be remembered that in spite of the horror with which a comparatively innocent girl may regard the jests and unveiled innuendoes of her companions, the corrupting influence of them in some degree remains.

There is another point to be considered. A young man or woman accused, and even convicted, on some petty charge, is not, therefore, given over irredeemably to crime. Punishment may have had the desired deterrent effect, and the individual in question resolves to walk in the ways of honesty henceforth. Let us take the case of a young servant-girl, who, on seeking a new situation, certainly does not mention any charge that has previously been brought against her. But by some accident she comes across some professional thief who recognises her as having been tried at certain assizes. He holds this knowledge as a Damocles' sword over her head, threatening to tell her employer of her past disgrace unless she keeps on friendly terms with him. These friendly terms often result in the disappearance of the employer's plate, and in the girl's becoming a member of the recognised criminal classes. The same thing may happen to a young apprentice who was once punished for stealing a few shillings from his master's till. Exposure to the influence and even to the eyes of a regular criminal is a danger that may far outweigh the salutary effects of punishment, especially with people whose moral nature one may reasonably assume to be too weak to resist the threats of exposing their past to which they are subjected.

Even where such evils do not result, there are many things connected with the removal of prisoners which are calculated to wound them needlessly, and also to brutalise the public. We quote the following facts from the report of the Howard Association: "Twelve men, some handcuffed, and others chained, were lately seen in the streets of Liverpool, waiting for a tramcar, on the roof of which they were then placed for conveyance to Kirkdale gaol, to the annoyance and pain of fellow passengers and bystanders. Again, in April, the Home Secretary admitted in Parliament that twenty prisoners, on the average, are daily marched through the streets of Durham. Then, in May, a report appeared in the newspapers of a prisoner condemned to death being jeered at by a crowd at a railway station in Cheshire, and addressed with such observations as 'This is your last journey,' etc. As to this particular case, it is but fair to state that the authorities in London deny the accuracy of the published statements. In June a complaint was made of a party of handcuffed prisoners travelling in a railway carriage with other passengers at Leeds. In July a tumultuous and almost riotous reception was accorded at the Shrewsbury Station to a prisoner under sentence of death." Such scenes can only be described as revolting, and every effort should be taken to prevent their continuance.

Another point demanding attention is the treatment of witnesses who are summoned occasionally from considerable distances to attend trials, and have sometimes to wait for several days before their evidence is called. To the poorer class of witnesses this is a great hardship. Their work is neglected, they may lose their situations by their prolonged absence, though their connection with the case may be of the most casual and innocent nature; but they have no redress for the losses involved by their attendance at the court. The committee appointed by the Home Secretary to enquire into this matter says that some of these, while waiting to be called, "are reduced to the utmost straits, and obliged to sell or pawn every article of clothing not absolutely necessary for actual decency before being released from attendance." Surely it would be possible to state in the summons an approximate date for the trial of the case for which their evidence is needed. It is very hard that they should have to hang about a strange town for a week to give evidence of a case which when called will be disposed of in an hour.

We have indicated work that remains to be done before our method of dealing with criminals can be regarded as satisfactory. Anyone desirous of obtaining fuller information on the subject can get it from the Secretary of the Howard Association, Mr. William Tallack, at 5, Bishopsgate Without, E.C.

